# A genome-wide association study reveals novel SNP markers associated with resilience traits in two Mediterranean dairy sheep breeds

**DOI:** 10.3389/fgene.2023.1294573

**Published:** 2023-11-22

**Authors:** Angeliki Argyriadou, Sofia Michailidou, Sotiria Vouraki, Valentina Tsartsianidou, Alexandros Triantafyllidis, Athanasios Gelasakis, Georgios Banos, Georgios Arsenos

**Affiliations:** ^1^ Laboratory of Animal Husbandry, School of Veterinary Medicine, Faculty of Health Sciences, Aristotle University of Thessaloniki, Thessaloniki, Greece; ^2^ Centre for Research and Technology Hellas, Institute of Applied Biosciences, Thessaloniki, Greece; ^3^ Department of Genetics, Development and Molecular Biology, School of Biology, Aristotle University of Thessaloniki, Thessaloniki, Greece; ^4^ Genomics and Epigenomics Translational Research (GENeTres), Center for Interdisciplinary Research and Innovation (CIRI-AUTH), Balkan Center, Thessaloniki, Greece; ^5^ Laboratory of Anatomy and Physiology of Farm Animals, Department of Animal Science, School of Animal Biosciences, Agricultural University of Athens, Athens, Greece; ^6^ Scotland’s Rural College, Easter Bush, Midlothian, United Kingdom

**Keywords:** dairy sheep, Chios breed, Frizarta breed, genome-wide association, resilience, milk somatic cell count, body condition score, lactation persistency

## Abstract

Genetic selection for higher productivity increased dairy sheep susceptibility to diseases and environmental stressors, challenging their health and welfare status and production efficiency. Improving resilience to such stressors can enhance their ability to face these challenges without compromising productivity. Our objective was to estimate genomic heritability and perform genome-wide association studies (GWAS) to detect SNPs and candidate genes associated with three proxy traits for resilience (milk somatic cell count—SCC, lactation persistency—LP, body condition score—BCS) of Chios and Frizarta dairy ewes. We used genome-wide genotypes of 317 Chios and 346 Frizarta ewes. Individual records of milk yield and BCS, and milk samples were collected monthly for two consecutive milking periods; samples were analyzed to determine SCC. The LP was calculated as the regression coefficient of daily milk yield on days from lambing. Within breed, variance components analyses and GWAS were performed using genomic relatedness matrices in single-trait animal linear mixed models. Genomic-based heritability estimates were relatively high (BCS: h^2^ = 0.54 and 0.55, SCC: h^2^ = 0.25 and 0.38, LP: h^2^ = 0.43 and 0.45, for Chios and Frizarta ewes, respectively), compared to previous pedigree-based studies. The GWAS revealed 7 novel SNPs associated with the studied traits; one genome-wide and two suggestive significant SNPs for SCC (Frizarta: rs403061409, rs424064526 and rs428540973, on chromosomes 9, 1 and 12, respectively), one suggestive significant SNP for BCS (Chios: rs424834097 on chromosome 4) and three suggestive significant SNPs for LP (Frizarta: rs193632931 and rs412648955 on chromosomes 1 and 6, Chios: rs428128299 on chromosome 3). Nineteen candidate genes were detected: two for BCS (Chios: *POT1*, *TMEM229A*), thirteen for SCC (Frizarta: *NTAQ1*, *ZHX1*, *ZHX2*, *LOC101109545*, *HAS2*, *DERL1*, *FAM83A*, *ATAD2*, *RBP7*, *FSTL1*, *CD80*, *HCLS1*, *GSK3B*) and four for LP (Frizarta: *GRID2*, *FAIM*, *CEP70*—Chios: *GRIP1*). Present results show that resilience in the studied dairy sheep breeds is heritable and advance existing knowledge on the genomic background of SCC, LP, and BCS. Future research will quantify effects of different alleles of significant SNPs on the studied traits and search for possible correlations among traits to facilitate their effective incorporation in breeding programs aiming to improve resilience.

## 1 Introduction

Dairy sheep production in the Mediterranean basin has been practiced over thousands of years and still constitutes an important part of the animal production sector with socioeconomic and cultural impact (Paraskevopoulou et al., 2020). A large variety of local dairy sheep breeds have evolved over the course of time driven by either natural or artificial selection processes, perfectly adapted to their local environment and traditional management practices. However, increasing demand for higher productivity led to intensification of production and strong selection pressure of sheep. In the previous decades, many local sheep breeds that failed to quickly adapt and adequately increase their milk production have been replaced by foreign improved ones (Argyriadou et al., 2020). Genetic selection focusing only on higher productivity adversely affected other important traits; improved dairy sheep breeds are likely to be less resilient to environmental stressors and more susceptible to diseases (Mucha et al., 2022). As a result, their health and welfare are easily compromised, affecting the overall efficiency of production. Resilience is defined as the animal’s ability to withstand challenging environmental stressors without compromising productivity (De Barbieri et al., 2023). The importance of animal resilience became more evident with the increasing incidence of climate change-related phenomena that further challenge the efficiency of farm animal production. Emergence of infectious diseases, aridification of grasslands, limited feedstock availability and heat/cold stress of the animals are consequences that underline the need for breeding more resilient animals (Cheng et al., 2022). In this context, the genetic potential of resilient local sheep breeds that are well-adapted to harsh environmental conditions and low-input rearing systems is gaining scientific interest (Dumont et al., 2020).

The genetic study of sheep resilience has so far involved diverse traits regarding resistance to disease, resilience to weather conditions, longevity and resource use efficiency (Sánchez-Molano et al., 2020; Tsartsianidou et al., 2021; Hine et al., 2022; Mucha et al., 2022; Machefert et al., 2023). In dairy sheep, resilience is mainly associated with good udder health combined with longevity, extended lactations and adequately high milk performance. Mastitis is one of the biggest health issues in dairy sheep farms with significant economic impact since it is associated with production loss, poor milk quality, high culling rate and compromised ewe welfare (Gelasakis et al., 2015). Immune response to intramammary infections induces inflammation characterized by neutrophil migration into the udder and increased epithelial cell apoptosis that lead to high milk somatic cell count (SCC). Increased milk SCC is typical of both clinical and subclinical mastitis, whereas low milk SCC is indicative of resilience to mastitis. Selecting for lower milk SCC, which is a heritable trait, succeeded in limiting mastitis incidence (Rupp et al., 2009). Therefore, genetic and genomic architecture of SCC is currently a major topic of scientific interest (Rupp et al., 2015; Banos et al., 2017; Sutera et al., 2021; Öner et al., 2021; Mohammadi et al., 2022). However, existing differences among populations as well as the polygenic inheritance of the trait complicate safe conclusions and breed-specific studies are necessary.

Lactation persistency (LP) is another important dairy trait, which represents the decline rate of milk yield following the peak of lactation; a lower rate is related to extended and more productive lactations (Stefanon et al., 2002). The observed decrease in milk yield as lactation progresses is associated with the rate of epithelial cell apoptosis in the mammary gland. Balancing intrinsic factors (innate regulating mechanisms, hormone excretion, reproductive stage, etc.), environmental parameters (photoperiod, milking type and frequency, etc.) and stressors (poor nutrition, mastitis, etc.) that affect cell death and proliferation processes, determines lactation length and persistency (Stefanon et al., 2002; Capuco et al., 2003; Pulina et al., 2007). Therefore, extended lactations may be indicative of dairy animals that are resilient to such environmental stressors. Lactation persistency is a heritable trait (Pulina et al., 2007), however genetic and especially genomic studies on dairy sheep are scarce (Pollott and Gootwine, 2001; Kominakis et al., 2002; Jonas et al., 2011; Carta et al., 2014).

Body condition scoring is a technique for subjectively assessing body fat reserves on live sheep. Since its conception in the 1960s it has become widely used because it is easily applicable on farm without the need for special equipment or handling, and foremost it is not biased by other tissue conformation and weight (Kenyon et al., 2014). The body condition scoring scale proposed by Russel et al. (1969) has been developed for meat sheep, however it is largely used for assessing body condition score (BCS) of dairy ewes and rams at different reproductive and lactation stages. Though BCS is a trait associated with productivity in meat sheep, it can be used as a proxy trait for resilience and efficiency in dairy sheep. Under similar nutritional management, energy balance and body fat reserves are expected to be relatively uniform among individuals of the same breed and production level; hence, deviation from an optimum BCS is an indicator of inefficient use of resources and poor resilience to environmental stressors. Although BCS is a trait of moderate heritability with improvement potential, relevant research data on dairy sheep are very limited (Machefert et al., 2023).

In the context of exploring the genetic potential for resilience in sheep breeds that are well-adapted to the local environment, the present study focuses on two highly productive local dairy sheep breeds, namely, Chios and Frizarta, reared under intensive and semi-intensive conditions in Greece. The two populations have never been under selection for resilience traits (Argyriadou et al., 2020). In the present study, we investigate the genomic basis underlying three proxy traits for resilience to disease and environmental stressors, namely, SCC, LP and BCS of Chios and Frizarta ewes. The objective of the study is twofold; i) to estimate heritability for the three resilience traits using genomic data and ii) to perform genome-wide association studies (GWAS) to detect SNP markers and candidate genes that are associated with the studied traits.

## 2 Materials and methods

### 2.1 Animals and farms

A total of 317 Chios and 346 Frizarta ewes were randomly selected from four farms (two per breed) located in northern and western Greece, respectively. Within each breed, one of the selected farms was managed intensively and the other one semi-intensively, so that the two most common sheep farming systems in Greece are represented. Selecting an equal number of representative flocks of both systems, eliminates introduction of biases related to system-specific farming practices and allows for a broader impact and practical implementation of results. Therefore, flocks were selected on the basis of being typical of the above farming systems and having the best management practices among members of the Chios Sheep Breeders’ Cooperative “Macedonia” and the Agricultural and Livestock Union of Western Greece. Ewes were fed roughages and a concentrate mix with the quantities being appropriately adjusted to meet their nutritional requirements, while they had *ad libitum* access to water. Furthermore, ewes in the two semi-intensively reared flocks were grazing during spring and summer months for 2–3 h daily in natural or cultivated grasslands. Details regarding the location and management practices of the farms are presented in Table 1.

**TABLE 1:** Farm locations and characteristics.

	Farm 1	Farm 2	Farm 3	Farm 4
Breed	Chios	Chios	Frizarta	Frizarta
Region	Thessaloniki	Kilkis	Agrinio	Agrinio
Altitude (m)	35	80	187	30
Rearing system	Intensive	Semi-intensive	Intensive	Semi-intensive
Milkings/day	2	2	3 in the first 2–3 months of lactation and 2 thereafter	3 in the first 2–3 months of lactation and 2 thereafter
Machine milking—Vacuum pressure (kPa)	40	37	38	39
Machine milking—Pulsation rate (/min)	150	150	150	150
Machine milking—Pulsation ratio	50:50	50:50	50:50	50:50
Grazing	No	Yes	No	Yes
Average milk production/Lactation (kg)	248.3	336.8	277.0	279.8

### 2.2 Data collection and sampling

Ewes were monitored once per month for two consecutive milking periods following the weaning of lambs at 42 days postpartum. Animal handling and recording was always performed by the same qualified veterinarian. Ewe BCS was assessed by palpation of the lumbar region using the methodology described by Russel et al. (1969). The scale had scores ranging from 1 (extreme emaciation) to 5 (obesity) with 0.25 increments. The milk yield of individual ewes was recorded monthly in a single milking using designated milk meters (Waikato^®^, New Zealand) attached to each milking unit, which enabled also the collection of individual milk samples (an amount of approximately 50 mL) using Falcon tubes (70 mL volume capacity). Milk samples were stored in a portable freezer at 4°C and transferred to the laboratory within 24 h to assess SCC using Fossomatic™ FC (Foss, Denmark). Blood samples were collected from the jugular vein once per ewe in vacuum tubes with anticoagulant factor (EDTA, BD Vacutainer^®^ Blood collection tubes, BD, United States) and stored at −20°C, until further processing.

### 2.3 Phenotypic data handling and quality control

A total of 3,856 monthly test milk yield records were collected of all ewes over both years of the study, corresponding to up to seven monthly records per ewe per milking period. Average milking lengths were 189 and 165 days for Chios and Frizarta ewes, respectively. Individual total daily milk yield was calculated from the recorded yield of one milking on each monthly recording occasion based on the following equation:

$$DMY=\left(\frac{TY}{\Delta t}\right)*1440$$ (1)

where **DMY** is the daily milk yield, **TY** is the recorded milk yield from one milking and **Δt** is the time period between the recorded and the previous milking (in minutes). The latter equation assumes that milk is produced evenly throughout the day and allows for calculating daily milk yield based on recording one milking per day when more than two milkings are implemented. It is used by the Chios Sheep Breeders’ Cooperative “Macedonia” for farms with high milking frequency, as an alternative to the standard methods suggested by the International Committee of Animal Recording (ICAR, 2018) that assume two milkings per day. In the present study, it was used to facilitate direct comparisons among animals since two of the farms performed three milkings per day in the first 2–3 months of the milking period.

Lactation persistency was then calculated for each year of the study, as the regression coefficient of daily milk yield (after peak of lactation) on days from lambing (Kominakis et al., 2002), according to the following linear model:

y=β*DIM+α (2)

where **y** is the daily milk yield, **DIM** is the fixed effect of days from lambing, **β** is the regression coefficient on DIM, representing LP and **α** is the intercept. The overall LP for the 2 years of the study was calculated as the average of the annual values and used for further analyses.

Individual repeated measurements of SCC (*n* = 3,239) were used to calculate the mean for each year of the study weighted over the respective daily milk yield, according to the following formula:

$$\bar{SCC}=\frac{\sum_{i=0}^{n}{DMY}_{i}*{SCC}_{i}}{\sum_{i=0}^{n}{DMY}_{i}}$$ (3)

where $\bar{\text{SCC}}$ is the annual weighted mean of SCC, **DMY**_**i**_ is the daily milk yield in the *i* ^th^ milk recording occasion and **SCC**_**i**_ is the respective SCC. The average of the weighted means of SCC for the 2 years of the study was considered for genomic analyses. In case ewes had records of SCC or LP in only 1 year of the study, the respective annual values were used. Ewes with only one SCC record per year were excluded. Due to deviations from the normal distribution, SCC data were logarithmically transformed prior to further analyses. The approach is a modified version of the methodology suggested by Rupp et al. (2003), which used milk somatic cell score weighted over days in milk.

Regarding BCS, all individual records (*n* = 3,784) were averaged over both years of the study. Poor body conformation was considered an indication of compromised health and welfare, which could possibly impact the expression of genetic potential (Kenyon et al., 2014); hence, ewes with average BCS less than 2 were excluded from the study (Munoz et al., 2019).

### 2.4 DNA extraction, animal genotyping and quality control

Genomic DNA was extracted from blood samples using the QIAamp DNA Mini and Blood Mini kit (QIAGEN, United States, RRID:SCR_008539), as previously described in detail by Tsartsianidou et al. (2021). DNA samples of Chios ewes were genotyped with the medium-density Illumina OvineSNP50 Genotyping Beadchip (Illumina Inc., San Diego, California, RRID:SCR_010233) which features 54,241 SNPs, whereas the updated Illumina OvineSNP50 Genotyping Beadchip v2 containing 53,516 SNPs was used for Frizarta samples.

Genotype quality control was performed with PLINK 1.9 software (RRID:SCR_001757; Purcell et al., 2007; Chang et al., 2015). All SNPs on non-autosomal regions were removed. Furthermore, SNPs with minor allele frequency (MAF) lower than 2%, call rate lower than 97% or deviating from Hardy-Weinberg equilibrium (HWE, *p*-value = 10^–6^) were filtered out. Sample call rate threshold was set at 90%. Chromosomal coordinates were allocated to SNPs based on the Oar_v4.0 genome assembly (The International Sheep Genomics Consortium, 2015; NCBI Assembly Archive Viewer, RRID:SCR_012917).

### 2.5 Principal component analysis (PCA) of genotypes

Principal Component Analysis (PCA) of animal genotypes was performed within breed using GEMMA software version 0.98.1 (Zhou and Stephens, 2012) to investigate possible population structures for subsequent analyses. The eigenvectors and eigenvalues of the decomposed matrices were then plotted in RStudio (RRID:SCR_000432; Posit team, 2022) with R version 4.1.2 (RRID:SCR_001905; R core team, 2021).

### 2.6 Estimation of genomic parameters

Variance components of LP, BCS and logarithmically transformed SCC were estimated within breed by residual maximum likelihood (REML) using a genomic relatedness matrix with the following single-trait animal linear mixed model:

y=Xτ+Zu+ε (4)

where **y** is the phenotypes vector, **τ** is the vector of fixed effects, **u** is the vector of random effects, **X** and **Z** are the design matrices that associate phenotypes with fixed and random effects, respectively, whereas **ε** is the vector of random residual errors. Preliminary analyses were performed to identify the fixed effects for the models. The tested variables included farm (2 levels for each breed), lactation number (in the first year of the study—6 levels, representing lactation number 1–5 and ≥6), recording years (2 levels, representing ewes with records in one or both years of the study), lambing season (3 levels—representing ewes lambing in 1: Summer-Autumn i.e., first lambing group of each year; 2: Winter-Spring i.e., second lambing group of each year; or 3: either first or second lambing group depending on the year) and prolificacy (covariate, representing the average prolificacy for both years of the study). Principal Components (PCs) from PCA accounting for measurable proportions of variance were also tested. Only variables with a statistically significant effect (*p* < 0.05) on each studied trait were retained in the final models of statistical analyses. The Akaike Information Criterion, the Bayesian Information Criterion and the magnitude of the residual variance were also considered as goodness-of-fit indicators, to compare tested models. The random additive genetic effect of ewe and residual effect were included in all models. In the Chios analyses, the model for BCS included lactation number, lambing season and the first two PCs as fixed effects; the model for SCC included lactation number and the first PC as fixed effects; the model for LP included farm and lambing season as fixed effects. In the Frizarta analyses, the model for BCS included farm and lambing season as fixed effects; respectively, farm and lactation number were included as fixed effects in the SCC model; the model for LP included the fixed effects of lambing season and recording years.

All data analyses were performed with ASReml software version 4.2 (Gilmour et al., 2021). The centered genomic relatedness matrix for each breed was created from post quality control genomic data with GEMMA software version 0.98.1 (Zhou and Stephens, 2012). Further formatting and inversion of the matrices was completed with R version 4.1.2 (RRID:SCR_001905; R core team, 2021) and package “AGHmatrix” (Amadeu et al., 2016). Finally, heritability for each trait was estimated as the ratio of the additive genetic to the total phenotypic variance. Statistical significance (*α* = 0.05) of heritability estimates was assessed based on the magnitude of the respective standard errors, with the two-tailed Student’s t-distribution.

### 2.7 Genome-wide association study

Within breed GWAS were conducted to identify SNPs associated with the studied traits. Model [4] was used in each case with the addition of **wβ**, where **w** is the vector of SNPs and **β** the associated effects. Furthermore, all GWAS models on Frizarta and Chios sheep traits included the first and first three PCs, respectively, to account for population structure. Genome-wide significance threshold was set at *p* < 0.05. A suggestive significance threshold was also set expecting one false positive association to occur once per GWAS (Duggal et al., 2008). After Bonferroni correction for multiple comparisons, the final genome-wide threshold values were *p* < 1.14E-06 and *p* < 1.05E-06 for Chios and Frizarta sheep, corresponding to the negative common logarithms (-log_10_) of 5.94 and 5.98. Corrected suggestive thresholds were *p* < 2.27E-05 and *p* < 2.11E-05 (corresponding to -log_10_ of 4.64 and 4.68) for Chios and Frizarta sheep, respectively. Analyses were performed with GEMMA software version 0.98.1 (Zhou and Stephens, 2012). Genome-wide plots of −log_10_(*p*-values) illustrating associations of SNPs with the studied traits and the respective Quantile-Quantile (Q-Q) plots for quality assessment of results were created in RStudio (RRID:SCR_000432; Posit team, 2022) with R version 4.1.2 (RRID:SCR_001905; R core team, 2021) and statistical R package “qqman” (Turner, 2018).

### 2.8 Gene annotation analysis

The NCBI Genome Data Viewer (RRID:SCR_002474, Rangwala et al., 2021) was used to determine the type of genomic regions in which the genome-wide and suggestive significant SNPs were located according to Oar_v4.0 genome assembly. Furthermore, we searched for candidate genes within regions of 1 Mb upstream and downstream of all significant SNPs, according to the levels of linkage disequilibrium reported by Tsartsianidou et al. (2021) and Kominakis et al. (2017) for the same Chios and Frizarta populations as the ones described here. AnimalQTLdb (RRID:SCR_001748; Hu et al., 2022) was used to investigate previously reported QTLs associated with the studied traits or located in common regions with the ones reported herein. To explore the biological functions of all annotated genes and the associated traits we used the NCBI Gene (RRID:SCR_002473), Ensembl release version 110 (RRID:SCR_002344; Cunningham et al., 2022) and UniProt (RRID:SCR_002380; The UniProt Consortium, 2023) databases and performed a literature review across species.

## 3 Results

### 3.1 Descriptive statistics and quality control

Descriptive statistics of phenotypic data after quality control are presented in Table 2.

**TABLE 2:** Descriptive statistics (number of observations and mean values) of body condition score (BCS), milk somatic cell count (SCC) and lactation persistency (LP) after quality control. n: number of observations; sd: standard deviation.

	BCS		SCC (cells/ml)		LP	
	n	Mean (±sd)	n	Mean (±sd)	n	Mean (±sd)
Chios	289	2.73 (±0.317)	288	263266 (±291899.5	293	−9.34 (±4.971)
Frizarta	299	2.62 (±0.276)	299	595116 (±873048.4)	298	−13.14 (±7.371)

A total of 10,243 and 6,065 SNPs were removed after quality control of Chios and Frizarta genotypes, respectively. Specifically for Chios genotypes, i) 2,157 SNPs were unmapped or located in non-autosomal regions, ii) 1,945 SNPs had call rate lower than 97%, iii) 236 deviated from HWE and iv) 5,905 had MAF lower than 2%; the respective numbers for Frizarta sheep were i) 2,144, ii) 1,999, iii) 244 and iv) 1,678 SNPs. The final datasets included 43,998 and 47,451 SNPs for Chios and Frizarta sheep, respectively. No samples were removed due to low call rate.

### 3.2 Population structure

Results of PCA on Chios and Frizarta genomic relatedness matrices are demonstrated in Supplementary Figures S1 and S2, respectively. Approximately 14% of the total variation observed in Chios population was explained by the first three PCs (Supplementary Figure S1A). Regarding the Frizarta population, the first PC accounted for circa 5.5% of the total variation, whereas all the other components individually accounted for less than 2.6% (Supplementary Figure S2A). Plotting the first and second or third PC revealed that population structure was associated with farm, in Chios population (Supplementary Figure S1B). Consequently, the first three PCs were considered in the following Chios sheep GWAS to account for population structure. In Frizarta, plotting the first and second PCs revealed population structure attributed to farm, as well (Supplementary Figure S2B). The first PC was fitted as covariate in the GWAS models for Frizarta sheep to adjust for population structure.

### 3.3 Genomic heritability

Heritability estimates for all the studied traits are presented in Table 3. High heritability estimates for BCS were observed (h^2^ = 0.54 and 0.55, for Chios and Frizarta sheep, respectively) and moderate to high for LP (h^2^ = 0.43 and 0.45) and SCC (h^2^ = 0.25 and 0.38). All estimates were statistically significant (*α* = 0.05), except for heritability of SCC in Chios sheep.

**TABLE 3:** Heritability estimates (± standard error in parentheses) of body condition score (BCS), milk somatic cell count (SCC) and lactation persistency (LP).

	BCS	SCC	LP
Chios	0.54 (±0.188)	0.25 (±0.251)	0.43 (±0.166)
Frizarta	0.55 (±0.174)	0.38 (±0.185)	0.45 (±0.172)

### 3.4 Genome-wide association study

**FIGURE 1:**
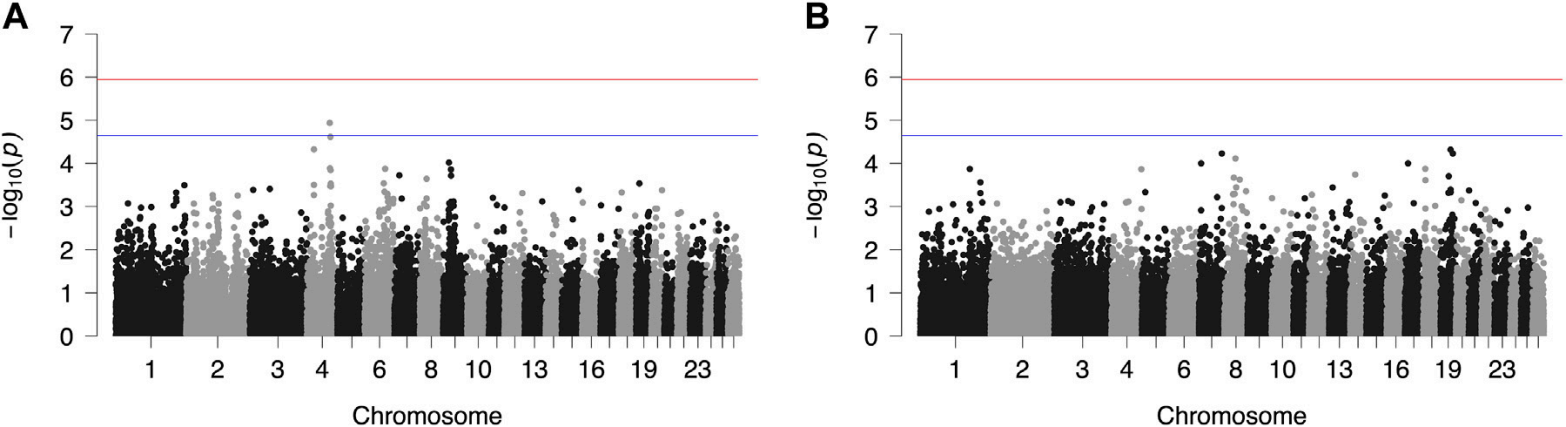
Genome-wide plots of −log_10_ (*p*-values) illustrating associations of single nucleotide polymorphisms (SNPs) with body condition score (BCS) of Chios **(A)** and Frizarta **(B)** sheep. Red and blue lines indicate the genome-wide and suggestive significance thresholds, respectively.

**FIGURE 2:**
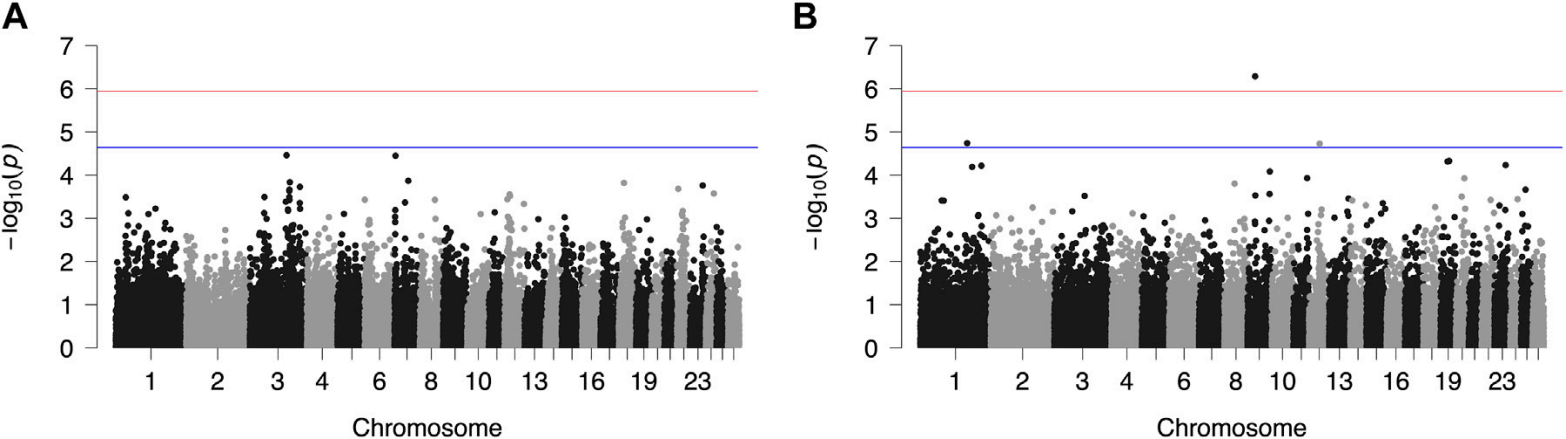
Genome-wide plots of −log_10_ (*p*-values) illustrating associations of single nucleotide polymorphisms (SNPs) with logarithmically transformed milk somatic cell count (SCC) of Chios **(A)** and Frizarta **(B)** sheep. Red and blue lines indicate the genome-wide and suggestive significance thresholds, respectively.

**FIGURE 3:**
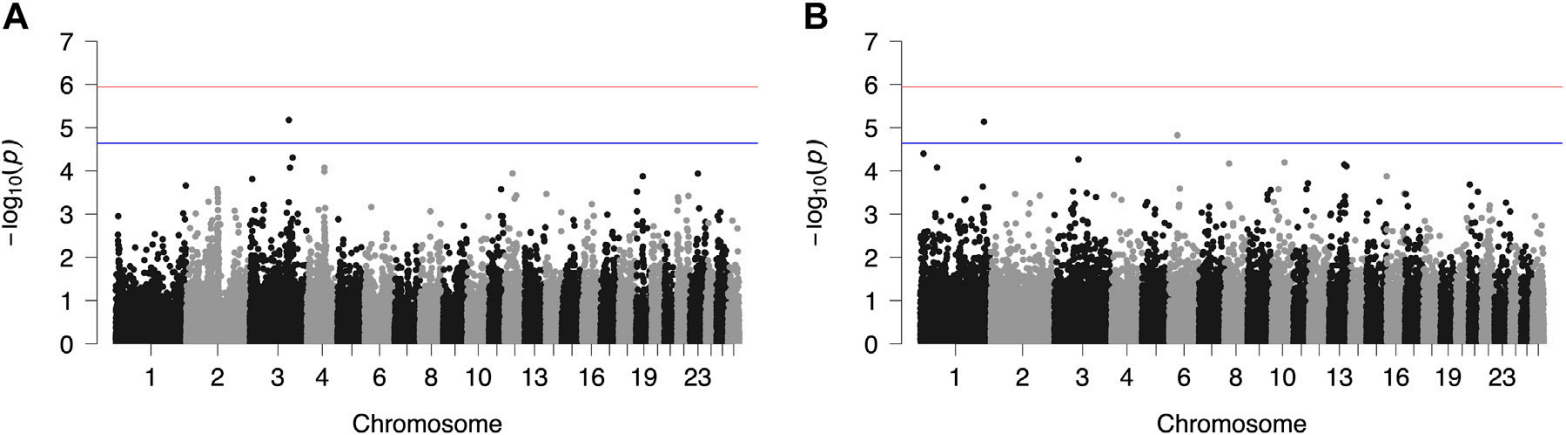
Genome-wide plots of −log_10_ (*p*-values) illustrating associations of single nucleotide polymorphisms (SNPs) with lactation persistency (LP) of Chios **(A)** and Frizarta **(B)** sheep. Red and blue lines indicate the genome-wide and suggestive significance thresholds, respectively.

Results from GWAS of BCS, SCC and LP are presented in Figures 1–3, respectively, and the corresponding Q-Q plots in Supplementary Figures S3–S5. Different genomic regions are involved in the inheritance of the studied traits in Chios and Frizarta populations; however, in all cases a polygenic mode of inheritance is implied. In total, one genome-wide and six suggestive significant SNPs were detected, two of the latter in Chios and four in Frizarta analyses (Table 4). Regarding LP, regions of interest were located on chromosomes 1 (rs193632931) and 6 (rs412648955) for Frizarta sheep and chromosome 3 (rs428128299) for Chios sheep. One genome-wide (rs403061409 on chromosome 9) and two suggestive significant SNPs (rs424064526 and rs428540973 on chromosomes 1 and 12, respectively) were associated with SCC in Frizarta sheep. In Chios, no significant SNP associations with SCC were detected, consistently with the non-significant heritability estimate for this trait; however, two SNPs on chromosomes 3 and 7 (rs416035680 and rs419886966, respectively) almost reached the suggestive significant threshold (*p*-value = 3.45E-05 and 3.54E-05, respectively). Finally, one suggestive significant SNP (rs424834097) located on chromosome 4 was associated with BCS in Chios sheep, whereas no significant associations were detected in Frizarta.

**TABLE 4:** Single nucleotide polymorphisms (SNPs) significantly associated with body condition score (BCS), milk somatic cell count (SCC) and lactation persistency (LP) at genome-wide (*p* < 1.14E-06 for Chios and *p* < 1.05E-06 for Frizarta sheep) and suggestive (*p* < 2.27E-05 for Chios and *p* < 2.11E-05 for Frizarta sheep) thresholds and genes located within 1 Mb upstream or downstream of the significant SNPs.

Trait	Breed	Chromosome	SNP	Position (bp)	−log_10_ (*p*-value)	Type of variant	Genes
SCC	Frizarta	9	rs403061409	30,159,232	6.29	Intergenic	WDYHV1 (NTAQ1), ATAD2, ZHX1, C9H8orf76, FAM83A, TBC1D31, ZHX2, LOC101110602, LOC101110341 (HAS2), DERL1, LOC101109545, LOC101110068
SCC	Frizarta	1	rs424064526	183,294,771	4.74	Intronic (Gene: FSTL1)	ARHGAP31, POGLUT1, TMEM39A, CD80, PLA1A, TIMMDC1, ADPRH, LOC105607786, LOC101107153, MAATS1 (CFAP91), POPDC2, NR1I2, GSK3B, GPR156, LRRC58, FSTL1, GTF2E1, HGD, NDUFB4, RABL3, STXBP5L, POLQ, GOLGB1, FBXO40, HCLS1, ARGFX
SCC	Frizarta	12	rs428540973	41,607,017	4.73	Intronic (Gene: RBP7)	EXOSC10, MASP2, TARDBP, SRM, CASZ1, PEX14, CORT, PGD, APITD1, DFFA, KIF1B, LOC105606447, UBE4B, RBP7, LZIC, NMNAT1, CTNNBIP1, PIK3CD, CLSTN1, TMEM201, SLC25A33, LOC105606444, LOC101113870, LOC105606443, SPSB1, LOC105616528, H6PD, GPR157, SLC2A5, CA6
LP	Frizarta	1	rs193632931	248,974,655	5.14	Intergenic	LOC105606041, FOXL2, PIK3CB, FAIM, CEP70, ESYT3, MRAS, NME9, ARMC8, DBR1, A4GNT, DZIP1L, CLDN18, LOC101112530, SOX14, LOC101111249
LP	Frizarta	6	rs412648955	32,299,373	4.83	Intronic (Gene: GRID2)	GRID2, CCSER1
LP	Chios	3	rs428128299	153,077,868	5.18	Intronic (Gene: GRIP1)	GRIP1, HELB, IRAK3, TMBIM4, LLPH, HMGA2, MSRB3, CAND1
BCS	Chios	4	rs424834097	89,155,631	4.94	Intronic (Gene: POT1)	POT1, GPR37, LOC101102641, LOC101102898, LOC101108219, LOC101108480, TMEM229A, LOC101102143, LOC101101889, LOC101107188, HYAL4 (LOC101106519)

### 3.5 Gene annotation analysis

The genome-wide significant SNP (rs403061409) associated with SCC in Frizarta sheep was located in an intergenic region approximately 530 kb from the nearest gene (*ZHX2*—zinc fingers and homeoboxes 2). Almost all suggestive significant SNPs were located within introns of protein coding genes. Specifically, *GRIP1* (glutamate receptor interacting protein 1) and *GRID2* (glutamate ionotropic receptor delta type subunit 2) genes included two SNPs, rs428128299 and rs412648955, associated with LP in Chios and Frizarta sheep, respectively. The rs424064526 and rs428540973, that were associated with SCC in Frizarta sheep, were located within *FSTL1* (follistatin like 1) and *RBP7* (retinol binding protein 7) genes, respectively. Last, rs424834097, associated with BCS in Chios sheep, was located within *POT1* (Protection of telomeres protein 1) gene. The only exception was rs193632931 associated with LP in Frizarta sheep, which was located in an intergenic region approximately 600 bp downstream of the nearest gene (*DZIP1L*—DAZ interacting zinc finger protein 1 like). According to the AnimalQTLdb, none of the significant SNPs were in regions of previously reported QTL associated with the studied traits in sheep. Within 1 Mb upstream or downstream of significant SNPs, a total of 105 genes were identified, 86 of which refer to Frizarta and 19 to Chios sheep; most of the genes were protein coding, 9 were pseudogenes and 4 were non-coding RNA genes (Supplementary Tables S1–S4).

## 4 Discussion

### 4.1 Genomic heritability

The present heritability estimates of SCC were higher than the ones reported in previous studies on the same or other dairy sheep breeds. Specifically, pedigree-based heritability estimates of SCC test-day records for Chios sheep were low to moderate and ranged from 0.09 to 0.18 depending on the stage of lactation at which records were collected (Ligda et al., 2003; Bramis et al., 2014; Banos et al., 2017). Slightly higher heritability estimates of 0.30–0.44 were reported by Psifidi et al. (2014) towards the end of lactation (between the 19th and 23rd week of lactation). To our knowledge, no studies have so far reported heritability estimates of SCC for Frizarta sheep. In other studies, heritability of somatic cell score (which is based on a binary logarithmic transformation of SCC) was also lower than the SCC heritability estimates presented here and ranged from 0.05 to 0.15 in Valle del Belice (Sutera et al., 2021), French Lacaune (Rupp et al., 2003) and blond-faced Manech sheep (Aguerre et al., 2022). However, heritability estimates of somatic cell score for Spanish Assaf and Sarda sheep (and their crosses with Lacaune) and of logarithmically transformed SCC for Churra sheep ranged from 0.21 to 0.28, similar to the present results for Chios sheep (Casu et al., 2010; Sánchez-Mayor et al., 2019; Pelayo et al., 2021). Nevertheless, it should be noted that in the present study SCC heritability estimate for Chios sheep had a high standard error and was not statistically significant (*α* = 0.05). Further investigation on a larger sample size may be necessary to allow safe conclusions regarding heritability of SCC in the studied Chios population.

Research data on heritability of BCS in dairy sheep are scarce. Aguerre et al. (2018) have reported BCS heritability estimates of 0.25 for blond-faced Manech sheep. The present estimates were also higher compared to the ones reported for meat and wool sheep, most of which ranged from 0.04 to 0.32 (Walkom and Brown, 2017; Macé et al., 2018; Snyman and Fisher, 2019; Marques et al., 2020; Oliveira et al., 2021; Rodrigues et al., 2021; Hickey et al., 2022; Ramos et al., 2023a). However, one study reported estimates of New Zealand Merino ewes up to 0.66 depending on the reproductive stage (Tait et al., 2018).

In the present study, heritability of LP lies close to the upper limit of previously reported estimates that ranged from 0.11 to 0.46, depending on the population and the definition of the trait which is largely inconsistent among studies (Pollott and Gootwine, 2001; Carta et al., 2014; Martinez Boggio et al., 2022). Different approaches for its estimation include calculating ratios of milk yield at different stages of lactation using either test-day or cumulative yields and estimation of specific parameters based on lactation curve modelling (Pulina et al., 2007). Kominakis et al. (2002) have used an approach similar to the present one and estimated heritability of 0.15 for Greek local Boutsko ewes, based on the regression coefficient expressing the milk yield decline across lactation.

Overall, the present heritability estimates were higher or similar to the highest estimates reported in the literature. However, in the present study, heritability of the studied traits was calculated based on genomic relatedness matrices of Chios and Frizarta populations instead of pedigree data which have been used in the above-mentioned studies. The accuracy of sheep pedigree has been questioned due to the specific characteristics and limitations of sheep rearing systems and reproduction practices that often result in false or missing parentage data. On the contrary, genomic relatedness matrices accurately capture family structure and relationships between animals (Hayes et al., 2009; Legarra et al., 2014), resulting in higher heritability estimates compared to pedigree-based approaches (Legarra et al., 2014; Cesarani et al., 2019). Differences regarding trait definitions, statistical models and analytical approaches may also contribute to the observed discrepancies among studies, as described above. Furthermore, deviating heritability estimates may reflect diverse inheritance patterns and genetic potential among sheep breeds, especially those used for different production purposes. Nevertheless, studied traits are heritable and therefore amenable to improvement through selective breeding. To facilitate inclusion in genetic improvement programs, further studies are needed to investigate possible favorable or antagonistic correlations between the studied and other important resilience or production traits.

### 4.2 Genome-wide association study and gene annotation

Population structure revealed by PCA was associated with the farm of origin of animals in both breeds, in accordance with the fact that targeted selection and mating practices is mainly performed on farm level. The effect was less intense in Frizarta sheep since artificial insemination is performed in some ewes (less than 30% of the flocks); in this case, the use of common sires between farms allows for more genetic admixture.

Previous GWAS for mastitis-related traits of different Mediterranean dairy sheep breeds have reported several SNPs and QTLs associated with SCC and somatic cell score, located on chromosomes 1–3, 7–8, 10, 16 and 19 (Rupp et al., 2015; Banos et al., 2017; Sutera et al., 2021; Öner et al., 2021), indicating a polygenic mode of inheritance and the involvement of different genes depending on the breed of study. For Chios sheep, Banos et al. (2017) reported associations of SCC with SNPs on chromosomes 2, 3, 16 and 19, unlike the present study that found none to be statistically significant. However, we found one SNP on chromosome 3 (rs416035680) that approached the suggestive significant threshold (*p* = 3.45E-05), indicating relevance to previous results of Banos et al. (2017). The main differences between the two studies are the mastitis customized 960-SNP array that was used by Banos et al. (2017), including SNPs on targeted chromosomes only, combined with the bigger sample size (*n* = 609) that increased statistical power. Similarly, no common regions with previously reported associations of SCC in Frizarta sheep were identified; Kominakis et al. (2019) have found significant SNPs on chromosomes 2, 18, 19 and 22. Methodological differences between studies may partly contribute to the discrepancy of results. However, a larger within breed genomic variability is suspected that may be captured in studies involving larger sample sizes. On chromosome 1, three significant SNPs for Valle del Belice sheep (Sutera et al., 2021) and one QTL for Spanish Churra sheep (Gutiérrez-Gil et al., 2018) have been associated with somatic cell score, consistently to present results for Frizarta sheep. Nevertheless, all were located far from the suggestive significant SNP (rs424064526) reported herein, the closest being circa 52 Mb apart. Furthermore, two QTLs on chromosome 9 were associated with somatic cell score (Mohammadi et al., 2022), one of which was located close (circa 500 kb) to the only genome-wide significant SNP (rs403061409 on chromosome 9) that was associated with SCC in Frizarta sheep, herein. Among genes found within 1 Mb of the latter SNP, *WDYHV1 (NTAQ1)*, *ZHX1* and *ZHX2* are involved in apoptosis and immune response pathways (Wang et al., 2009; Ma et al., 2016). Furthermore, the identified *LOC101109545* gene which encodes the 60S ribosomal protein L13a, is also involved in apoptosis and plays a crucial role in regulating inflammation through its expression in macrophages (Poddar et al., 2013). Moreover, *LOC101110341* (*HAS2*) is involved in synthesis of hyaluronan, which is associated with mammary growth during gestation (Tolg et al., 2017). The latter gene along with *ZHX2*, *DERL1*, *FAM83A* and *ATAD2* have been associated with intense symptoms, resistance to chemotherapy and bad prognosis of human breast cancer and metastatic canine mammary adenocarcinomas, implying interference in mammary cell growth and immune regulation (Kalashnikova et al., 2010; Klopfleisch et al., 2010; Lee et al., 2012; Fang et al., 2021; Choi et al., 2022). In accordance with the above, the suggestive significant SNP rs428540973 on chromosome 12, which was associated with SCC in Frizarta sheep, is located within *RBP7*; downregulation of its expression in human breast cancer has been associated with bad prognosis (Lin et al., 2022). Interestingly, the suggestive SNP for SCC on chromosome 1, rs424064526, is located within the intronic region of *FSTL1*, which is involved in pathogenetic pathways promoting tissue inflammation and subsequent fibrosis (Andreichenko et al., 2019; Li et al., 2021). Other possibly relevant genes located within 1 Mb around the above suggestive significant SNPs are *CD80*, *HCLS1*, *GSK3B*. The first of these genes encodes the cluster differentiation 80 protein, which plays an important role in T-cell migration to the mammary gland during infection (Bharathan and Mullarky, 2011). Similarly, leukocyte migration is also regulated by hematopoietic cell-specific lyn-substrate 1, an actin-binding protein encoded by *HCLS1*. Moreover, the latter protein is involved in differentiation of myeloid cells and cases of underexpression have been associated with human congenital neutropenia (Samareh-Abolhasani et al., 2013; Castro-Ochoa et al., 2018). Finally, *GSK3B* is indirectly involved in regulating mammary cell multiplication, since it affects the activation process of other proteins that are responsible for cell cycle regulation (Musumeci et al., 2015). In dairy sheep, high SCC is associated with mastitis (either clinical or subclinical), which is caused by the immune response of the mammary gland to intramammary pathogens leading to increased cell apoptosis, udder inflammation and fibrosis (Gelasakis et al., 2015). Considering the physiological function of the above genes and their role in mammary pathogenesis or resistance to disease, we suggest that they may participate in SCC regulation.

Research data on the genomic basis of LP in dairy sheep are scarce; to our knowledge, only one study has reported a relevant QTL on chromosome 11 for Awassi × Merino dual purpose crosses (Jonas et al., 2011). In the present study, suggestive significant SNP rs412648955 affecting LP of Frizarta ewes was located on chromosome 6, in the intronic region of *GRID2*. This gene is mainly expressed in the cerebellum and has been associated with gonadotropin secretion from pituitary gland and reproductive traits in sheep and cattle (Sugimoto et al., 2010; Chen et al., 2022), and secretion of GnRH in female mice (Todman et al., 2005). Interestingly, a photoperiodically regulated effect of GnRH, inducing prolactin secretion, has been observed in ewes and mares (Henderson et al., 2008). The above observations, collectively, suggest a possible association between *GRID2* expression and milk production and persistency. Another study (Nazar et al., 2022) reported association of *GRID2* with conformation of the udder central suspensory ligament in cows; poor udder conformation is often related to incomplete milking, which increases the risk of mastitis and reduces milk production, thereby negatively affecting LP. Closely located to the suggestive significant SNP rs193632931 associated with LP in Frizarta sheep (chromosome 1), *FAIM* gene is involved in processes inhibiting cell apoptosis and its expression in sheep skin has been associated with superfine wool quality (Tian et al., 2014); expression in the mammary gland may have a protective effect against apoptosis, therefore improving LP (Capuco et al., 2003). Another candidate gene in the same region is *CEP70*. Hao et al. (2020) reported expression of *CEP70* gene in the mammary gland of two local Chinese sheep breeds. The differentially lower expression level in the breed with the highest milk production indicates a possible adverse effect of *CEP70* expression on milk production and persistency. For Chios sheep, the suggestive significant SNP associated with LP, rs428128299 on chromosome 3, was located in the intronic region of *GRIP1*, a gene with significant expression in the uterus of sheep and cattle during oestrus (Hlaing et al., 2001). Considering the adverse effect of oestrus on milk production (King, 1977; Dobson et al., 2007), a possible indirect effect of *GRIP1* on LP could be underlying the latter association.

Previously reported QTLs associated with BCS of meat and wool sheep are located on multiple chromosomes across the genome. All studies reported regions on chromosomes 1, 2 and 10 (Amorim et al., 2018; Macé et al., 2022; Ramos et al., 2023b). In addition, Amorim et al. (2018) reported a relevant QTL on chromosome 4. In the present study, the suggestive significant SNP associated with BCS in Chios sheep (rs424834097) located on chromosome 4 was not included in the QTL region in the study of Amorim et al. (2018). The observed differences compared to the present results probably reflect the distinctive body conformation and body fat mobilization processes among dairy and other types of sheep. The rs424834097 SNP is located within the intronic region of *POT1*, which encodes a protein that regulates telomere length and preserves the overall integrity of chromosomal DNA. Telomere length is reduced under oxidative stress conditions; therefore, it is considered an indicator of cell aging. Metabolic stress throughout lactation in dairy cows, obesity in women and poor body condition of female mink have been related to shorter telomere length (Valdes et al., 2005; Moreno-Navarrete et al., 2010; Boudreau et al., 2014; Laubenthal et al., 2016), supporting the present findings that associate BCS with *POT1*. *TMEM229A* gene, which is located in the proximity of rs424834097, has been previously associated with body conformation traits in cattle and high-performance phenotypes in athletes (Boulygina et al., 2020; Abdalla et al., 2021). While mechanisms underlying the latter observations are not fully elucidated, results point to the possibility of *TMEM229A* being a candidate gene for BCS.

Overall, the results of the present study indicate that resilience traits of highly productive dairy ewes are heritable and report 7 novel SNP markers and 19 candidate genes for BCS (rs424834097 and *POT1*, *TMEM229A*), SCC (rs403061409, rs424064526, rs428540973 and *NTAQ1*, *ZHX1*, *ZHX2*, *LOC101109545*, *HAS2*, *DERL1*, *FAM83A*, *ATAD2*, *RBP7*, *FSTL1*, *CD80*, *HCLS1*, *GSK3B*) and LP (rs193632931, rs412648955, rs428128299 and *GRID2*, *FAIM*, *CEP70*, *GRIP1*) of Chios and Frizarta ewes, on chromosomes 1, 3, 4, 6, 9 and 12. The absence of common SNPs or genes between the two breeds and discrepancies from previously reported genomic regions associated with the studied traits in other breeds, underline their polygenic inheritance and the need for breed-specific studies that will facilitate the effective incorporation of such traits in breeding programs. The present study is expected to expand existing knowledge regarding the inheritance of such traits and contribute towards breeding more resilient animals. Studies on a larger sample size would be beneficial to confirm our findings. Further investigation of the present findings will focus on the different alleles of significant SNPs and quantification of their genetic contribution and impact on the associated traits. Moreover, possible correlations among the studied and other important production traits constitute another key aspect that needs to be investigated. Such studies will facilitate identification and selection of favorable alleles, and inclusion of the studied traits as objectives in breeding programs aiming to improve resilience without compromising productivity.

## Data Availability

The datasets presented in this study can be found in online repositories. The names of the repository/repositories and accession number(s) can be found below: https://zenodo.org/deposit/8344546, DOI: 10.5281/zenodo.8344546.

## Abbreviations

SNP, Single nucleotide polymorphism; BCS, Body condition score; SCC, Somatic cell count; LP, Lactation persistency; GWAS, Genome-wide association study; PCA, Principal component analysis; PC, Principal component; MAF, Minor allele frequency; HWE, Hardy-Weinberg equilibrium.
