# Supplementary material for: A genome-wide association study reveals novel SNP markers associated with resilience traits in two Mediterranean dairy sheep breeds
Source: Front Genet. 2023 Nov 22;14:1294573. doi: 10.3389/fgene.2023.1294573 (PMC10702769; doi:10.3389/fgene.2023.1294573)
Supplement: Supplementary file 2 [file Table1.DOCX]

Supplementary Material

# Supplementary Tables

**Supplementary Table 1.** Details of genes located within 1Mb upstream and downstream of the genome-wide and suggestive significant single nucleotide polymorphisms (SNPs) associated with body condition score (BCS) in Chios ewes.

| OAR | SNP | -log_10_  (p-value) | Ensembl Gene ID | Gene Name | Type | Description |
| --- | --- | --- | --- | --- | --- | --- |
| 4 | rs424834097 | 4.94 | ENSOARG00020004263 | POT1 | protein coding | protection of telomeres 1 [Source: NCBI gene; Gene ID:101107695] |
|  |  |  | ENSOARG00020004225 | GPR37 | protein coding | G protein-coupled receptor 37 [Source: NCBI gene; Gene ID:101107437] |
|  |  |  | NA | LOC101102641 | pseudogene | developmentally-regulated GTP-binding protein 1-like [Source: NCBI gene; Gene ID:101102641] |
|  |  |  | NA | LOC101102898 | pseudogene | lys-63-specific deubiquitinase BRCC36-like [Source: NCBI gene; Gene ID:101102898] |
|  |  |  | NA | LOC101108219 | pseudogene | elongation factor 1-alpha 1-like [Source: NCBI gene; Gene ID: 101108219] |
|  |  |  | NA | LOC101108480 | pseudogene | 40S ribosomal protein S3a-like [Source: NCBI gene; Gene ID: 101108480] |
|  |  |  | NA | TMEM229A | protein coding | transmembrane protein 229A [Source: NCBI gene; Gene ID: 101102388] |
|  |  |  | ENSOARG00020004171 | LOC101102143 | protein coding | hyaluronidase PH-20-like [Source: NCBI gene; Gene ID: 101102143] |
|  |  |  | NA | LOC101101889 | protein coding | hyaluronidase-4-like [Source: NCBI gene; Gene ID: 101101889] |
|  |  |  | NA | LOC101107188 | protein coding | hyaluronidase PH-20 [Source: NCBI gene; Gene ID: 101107188] |
|  |  |  | ENSOARG00020004017 | HYAL4 (LOC101106519) | protein coding | hyaluronidase 4 [Source: NCBI gene; Gene ID: 101106519] |
