# Supplementary material for: A genome-wide association study reveals novel SNP markers associated with resilience traits in two Mediterranean dairy sheep breeds
Source: Front Genet. 2023 Nov 22;14:1294573. doi: 10.3389/fgene.2023.1294573 (PMC10702769; doi:10.3389/fgene.2023.1294573)
Supplement: Supplementary file 4 [file Image2.PDF]

Supplementary Material

1 Supplementary Figures

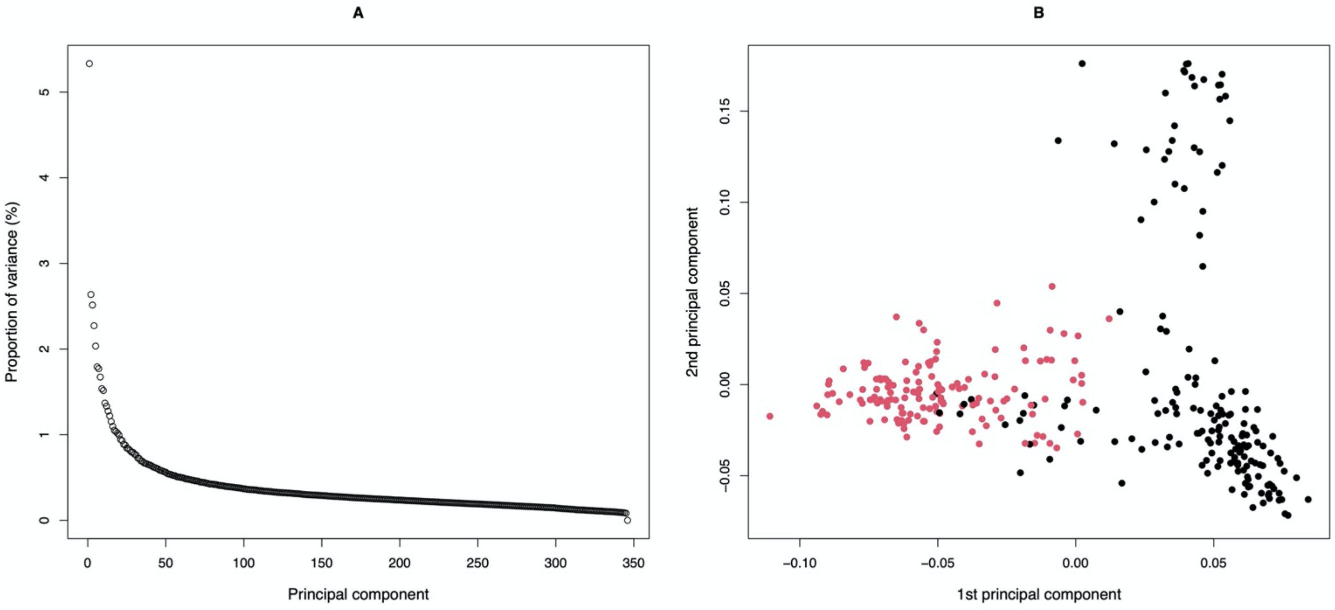

Supplementary Figure 2. Results of the principal component analysis (PCA) for Frizarta sheep. **(A)** Proportion of variation (%) corresponding to each principal component of the decomposed genomic relatedness matrix. **(B)** Plot of the first and second principal components illustrating the population structure attributed to the farm effect.
