# Supplementary material for: A genome-wide association study reveals novel SNP markers associated with resilience traits in two Mediterranean dairy sheep breeds
Source: Front Genet. 2023 Nov 22;14:1294573. doi: 10.3389/fgene.2023.1294573 (PMC10702769; doi:10.3389/fgene.2023.1294573)
Supplement: Supplementary file 5 [file Image3.PDF]

Supplementary Material

1 Supplementary Figures

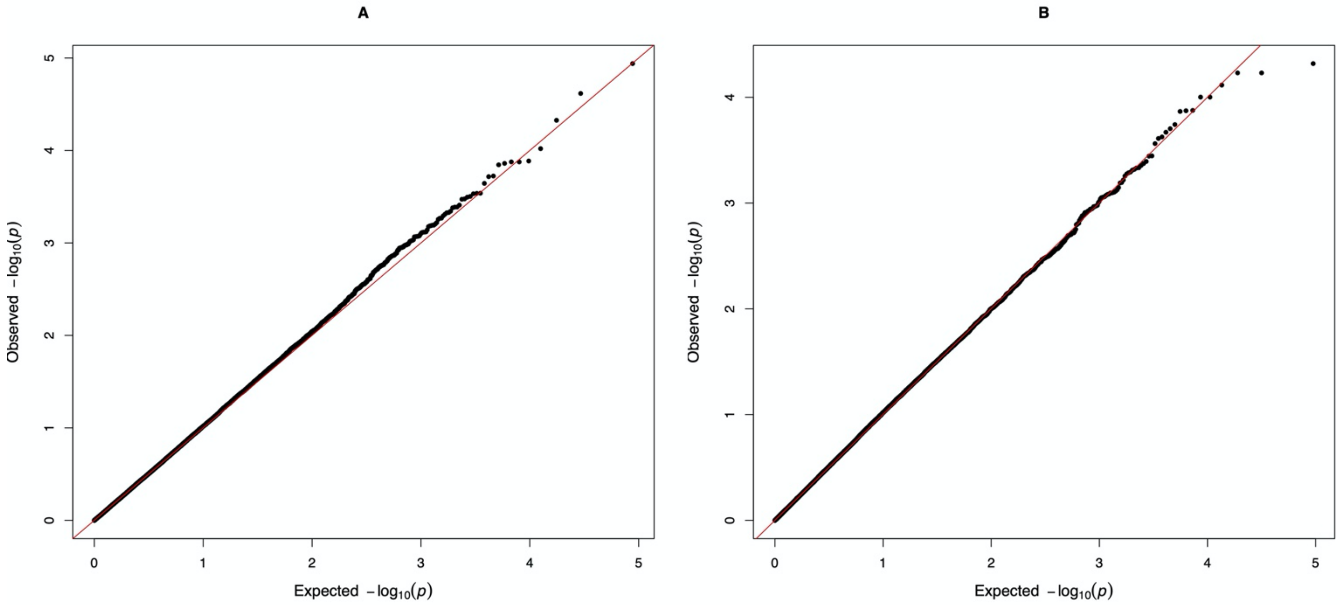

Supplementary Figure 3. Q-Q plot of the expected vs the observed distribution of *P*-values for body condition score (BCS) of Chios **(A)** and Frizarta **(B)** sheep.
