# Supplementary material for: A genome-wide association study reveals novel SNP markers associated with resilience traits in two Mediterranean dairy sheep breeds
Source: Front Genet. 2023 Nov 22;14:1294573. doi: 10.3389/fgene.2023.1294573 (PMC10702769; doi:10.3389/fgene.2023.1294573)
Supplement: Supplementary file 6 [file Table2.DOCX]

Supplementary Material

# Supplementary Tables

**Supplementary Table 2.** Details of genes located within 1Mb upstream and downstream of the genome-wide and suggestive significant single nucleotide polymorphisms (SNPs) associated with lactation persistency (LP) in Chios ewes.

| OAR | SNP | -log_10_  (p-value) | Ensembl Gene ID | Gene Name | Type | Description |
| --- | --- | --- | --- | --- | --- | --- |
| 3 | rs428128299 | 5.18 | ENSOARG00020005765 | GRIP1 | protein coding | glutamate receptor interacting protein 1 [Source: NCBI gene; Gene ID:101115022] |
|  |  |  | ENSOARG00020006202 | HELB | protein coding | DNA helicase B [Source: NCBI gene; Gene ID:101108210] |
|  |  |  | ENSOARG00020006254 | IRAK3 | protein coding | interleukin 1 receptor associated kinase 3 [Source: NCBI gene; Gene ID:101115273] |
|  |  |  | ENSOARG00020006439 | TMBIM4 | protein coding | transmembrane BAX inhibitor motif containing 4 [Source: NCBI gene; Gene ID: 101115532] |
|  |  |  | ENSOARG00020038075 | LLPH | protein coding | LLP homolog, long-term synaptic facilitation factor [Source: NCBI gene; Gene ID: 101108472] |
|  |  |  | NA | HMGA2 | protein coding | high mobility group AT-hook 2 [Source: NCBI gene; Gene ID: 105609946] |
|  |  |  | ENSOARG00020029129 | MSRB3 | protein coding | methionine sulfoxide reductase B3 [Source: NCBI gene; Gene ID: 101108734] |
|  |  |  | ENSOARG00020005522 | CAND1 | protein coding | cullin associated and neddylation dissociated 1 [Source: NCBI gene; Gene ID: 101107775] |
