# Supplementary material for: A genome-wide association study reveals novel SNP markers associated with resilience traits in two Mediterranean dairy sheep breeds
Source: Front Genet. 2023 Nov 22;14:1294573. doi: 10.3389/fgene.2023.1294573 (PMC10702769; doi:10.3389/fgene.2023.1294573)
Supplement: Supplementary file 7 [file Table3.DOCX]

Supplementary Material

# Supplementary Tables

**Supplementary Table 3.** Details of genes located within 1Mb upstream and downstream of the genome-wide and suggestive significant single nucleotide polymorphisms (SNPs) associated with lactation persistency (LP) in Frizarta ewes.

| OAR | SNP | -log10  (p-value) | Ensembl Gene ID | Gene Name | Type | Description |
| --- | --- | --- | --- | --- | --- | --- |
| 1 | rs193632931 | 5.14 | ENSOARG00020010654 | LOC105606041 | protein coding | proline-rich protein 23B-like [Source: NCBI gene; Gene ID: 105606041] |
|  |  |  | ENSOARG00020010684 | FOXL2 | protein coding | forkhead box L2 [Source: NCBI gene; Gene ID: 106990809] |
|  |  |  | ENSOARG00020010835 | PIK3CB | protein coding | phosphatidylinositol-4,5-bisphosphate 3-kinase catalytic subunit beta [Source: NCBI gene; Gene ID: 101109759] |
|  |  |  | ENSOARG00020011065 | FAIM | protein coding | Fas apoptotic inhibitory molecule [Source: NCBI gene; Gene ID: 101110017] |
|  |  |  | ENSOARG00020011206 | CEP70 | protein coding | centrosomal protein 70 [Source: NCBI gene; Gene ID: 101111770] |
|  |  |  | ENSOARG00020011695 | ESYT3 | protein coding | extended synaptotagmin 3 [Source: NCBI gene; Gene ID: 101112022] |
|  |  |  | ENSOARG00020012177 | MRAS | protein coding | muscle RAS oncogene homolog [Source: NCBI gene; Gene ID: 101110290] |
|  |  |  | ENSOARG00020012267 | NME9 | protein coding | NME/NM23 family member 9 [Source: NCBI gene; Gene ID: 101110896] |
|  |  |  | ENSOARG00020012965 | ARMC8 | protein coding | armadillo repeat containing 8 [Source: NCBI gene; Gene ID: 101111147] |
|  |  |  | ENSOARG00020014988 | DBR1 | protein coding | debranching RNA lariats 1 [Source: NCBI gene; Gene ID: 101111415] |
|  |  |  | ENSOARG00020015112 | A4GNT | protein coding | alpha-1,4-N-acetylglucosaminyltransferase [Source: NCBI gene; Gene ID: 101111851] |
|  |  |  | ENSOARG00020015148 | DZIP1L | protein coding | DAZ interacting zinc finger protein 1 like [Source: NCBI gene; Gene ID: 101112535] |
|  |  |  | ENSOARG00020015263 | CLDN18 | protein coding | claudin 18 [Source: NCBI gene; Gene ID: 101112106] |
|  |  |  | ENSOARG00020015410 | LOC101112530 | pseudogene | phosphoglycerate mutase 1-like [Source: NCBI gene; Gene ID: 101112530] |
|  |  |  | ENSOARG00020015428 | SOX14 | protein coding | SRY-box transcription factor 14 [Source: NCBI gene; Gene ID: 101112787] |
|  |  |  | NA | LOC101111249 | pseudogene | trafficking protein particle complex subunit 1-like [Source: NCBI gene; Gene ID: 101111249] |
| 6 | rs412648955 | 4.83 | ENSOARG00020021077 | GRID2 | protein coding | glutamate ionotropic receptor delta type subunit 2 [Source: NCBI gene; Gene ID:101117078] |
|  |  |  | ENSOARG00020021891 | CCSER1 | protein coding | coiled-coil serine rich protein 1 [Source: NCBI gene; Gene ID:101117333] |
