# Supplementary material for: A genome-wide association study reveals novel SNP markers associated with resilience traits in two Mediterranean dairy sheep breeds
Source: Front Genet. 2023 Nov 22;14:1294573. doi: 10.3389/fgene.2023.1294573 (PMC10702769; doi:10.3389/fgene.2023.1294573)
Supplement: Supplementary file 8 [file Table4.DOCX]

Supplementary Material

# Supplementary Tables

**Supplementary Table 4.** Details of genes located within 1Mb upstream and downstream of the genome-wide and suggestive significant single nucleotide polymorphisms (SNPs) associated with milk somatic cell count (SCC) in Frizarta ewes.

| OAR | SNP | -log10  (p-value) | Ensembl Gene ID | Gene Name | Type | Description |
| --- | --- | --- | --- | --- | --- | --- |
| 9 | rs403061409 | 6.29 | ENSOARG00020015073 | WDYHV1 (NTAQ1) | protein coding | N-terminal glutamine amidase 1 [Source: NCBI gene; Gene ID: 101103653] |
|  |  |  | ENSOARG00020015273 | ATAD2 | protein coding | ATPase family AAA domain containing 2 [Source: NCBI gene; Gene ID: 101103903] |
|  |  |  | ENSOARG00020015701 | ZHX1 | protein coding | zinc fingers and homeoboxes 1 [Source: NCBI gene; Gene ID: 101108317] |
|  |  |  | ENSOARG00020015718 | C9H8orf76 | protein coding | chromosome 9 C8orf76 homolog [Source: NCBI gene; Gene ID: 101108578] |
|  |  |  | ENSOARG00020015775 | FAM83A | protein coding | family with sequence similarity 83 member A [Source: NCBI gene; Gene ID: 101108840] |
|  |  |  | ENSOARG00020015866 | TBC1D31 | protein coding | TBC1 domain family member 31 [Source: NCBI gene; Gene ID: 101104158] |
|  |  |  | ENSOARG00020016397 | ZHX2 | protein coding | zinc fingers and homeoboxes 2 [Source: NCBI gene; Gene ID: 101109810] |
|  |  |  | ENSOARG00020016454 | LOC101110602 | protein coding | hyaluronan synthase 2-like [Source: NCBI gene; Gene ID: 101110602] |
|  |  |  | ENSOARG00020028617 | LOC101110341 (HAS2) | protein coding | hyaluronan synthase 2 [Source: NCBI gene; Gene ID: 101110341] |
|  |  |  | ENSOARG00020029281 | DERL1 | protein coding | derlin 1 [Source: NCBI gene; Gene ID: 100037686] |
|  |  |  | NA | LOC101109545 | protein coding | 60S ribosomal protein L13a [Source: NCBI gene; Gene ID: 101109545] |
|  |  |  | NA | LOC101110068 | pseudogene | 40S ribosomal protein S27-like [Source: NCBI gene; Gene ID: 101110068] |
| 1 | rs424064526 | 4.74 | ENSOARG00020001802 | ARHGAP31 | protein coding | Rho GTPase activating protein 31 [Source: NCBI gene; Gene ID: 101105202] |
|  |  |  | ENSOARG00020001870 | POGLUT1 | protein coding | protein O-glucosyltransferase 1 [Source: NCBI gene; Gene ID: 101105708] |
|  |  |  | ENSOARG00020001841 | TMEM39A | protein coding | transmembrane protein 39A [Source: NCBI gene; Gene ID: 101105459] |
|  |  |  | ENSOARG00020001923 | CD80 | protein coding | CD80 molecule [Source: NCBI gene; Gene ID: 554317] |
|  |  |  | ENSOARG00020001966 | PLA1A | protein coding | phospholipase A1 member A [Source: NCBI gene; Gene ID: 101106480] |
|  |  |  | ENSOARG00020001894 | TIMMDC1 | protein coding | translocase of inner mitochondrial membrane domain containing 1 [Source: NCBI gene; Gene ID: 101105967] |
|  |  |  | ENSOARG00020001952 | ADPRH | protein coding | ADP-ribosylarginine hydrolase [Source: NCBI gene; Gene ID:101106222] |
|  |  |  | NA | LOC105607786 | ncRNA | uncharacterized LOC105607786 [Source: NCBI gene; Gene ID:105607786] |
|  |  |  | ENSOARG00020002013 | LOC101107153 | protein coding | cytochrome c oxidase copper chaperone [Source: NCBI gene; Gene ID:101107153] |
|  |  |  | ENSOARG00020002045 | MAATS1 (CFAP91) | protein coding | cilia and flagella associated protein 91 [Source: NCBI gene; Gene ID:101107403] |
|  |  |  | ENSOARG00020001991 | POPDC2 | protein coding | popeye domain containing 2 [Source: NCBI gene; Gene ID:101106898] |
|  |  |  | ENSOARG00020002112 | NR1I2 | protein coding | nuclear receptor subfamily 1 group I member 2 [Source: NCBI gene; Gene ID:101118075] |
|  |  |  | ENSOARG00020002145 | GSK3B | protein coding | glycogen synthase kinase 3 beta [Source: NCBI gene; Gene ID:100169938] |
|  |  |  | ENSOARG00020002267 | GPR156 | protein coding | G protein-coupled receptor 156 [Source: NCBI gene; Gene ID:101107652] |
|  |  |  | ENSOARG00020031516 | LRRC58 | protein coding | leucine rich repeat containing 58 [Source: NCBI gene; Gene ID:101108095] |
|  |  |  | ENSOARG00020002313 | FSTL1 | protein coding | follistatin like 1 [Source: NCBI gene; Gene ID:101118330] |
|  |  |  | ENSOARG00020002411 | GTF2E1 | protein coding | general transcription factor IIE subunit 1 [Source: NCBI gene; Gene ID:101109136] |
|  |  |  | ENSOARG00020002382 | HGD | protein coding | homogentisate 1,2-dioxygenase [Source: NCBI gene; Gene ID:101108617] |
|  |  |  | ENSOARG00020002367 | NDUFB4 | protein coding | NADH:ubiquinone oxidoreductase subunit B4 [Source: NCBI gene; Gene ID:101108353] |
|  |  |  | ENSOARG00020002395 | RABL3 | protein coding | RAB, member of RAS oncogene family like 3 [Source: NCBI gene; Gene ID:101108873] |
|  |  |  | ENSOARG00020002489 | STXBP5L | protein coding | syntaxin binding protein 5L [Source: NCBI gene; Gene ID:101109405] |
|  |  |  | ENSOARG00020003708 | POLQ | protein coding | DNA polymerase theta [Source: NCBI gene; Gene ID:101109663] |
|  |  |  | ENSOARG00020004009 | GOLGB1 | protein coding | golgin B1 [Source: NCBI gene; Gene ID:101110468] |
|  |  |  | ENSOARG00020003874 | FBXO40 | protein coding | F-box protein 40 [Source: NCBI gene; Gene ID:101109929] |
|  |  |  | ENSOARG00020003906 | HCLS1 | protein coding | hematopoietic cell-specific Lyn substrate 1 [Source: NCBI gene; Gene ID:101110196] |
|  |  |  | NA | ARGFX | protein coding | arginine-fifty homeobox [Source: NCBI gene; Gene ID:101118848] |
| 12 | rs428540973 | 4.73 | ENSOARG00020014377 | EXOSC10 | protein coding | exosome component 10 [Source: NCBI gene; Gene ID:101113610] |
|  |  |  | ENSOARG00020014599 | MASP2 | protein coding | MBL associated serine protease 2 [Source: NCBI gene; Gene ID:101112586] |
|  |  |  | ENSOARG00020014777 | TARDBP | protein coding | TAR DNA binding protein [ Ovis aries (sheep) ] [Source: NCBI gene; Gene ID:101114038] |
|  |  |  | ENSOARG00020014558 | SRM | protein coding | spermidine synthase [Source: NCBI gene; Gene ID:101112338] |
|  |  |  | ENSOARG00020014921 | CASZ1 | protein coding | castor zinc finger 1 [Source: NCBI gene; Gene ID:101112837] |
|  |  |  | ENSOARG00020015026 | PEX14 | protein coding | peroxisomal biogenesis factor 14 [Source: NCBI gene; Gene ID:101114292] |
|  |  |  | ENSOARG00020015142 | CORT | protein coding | cortistatin [Source: NCBI gene; Gene ID:100233238] |
|  |  |  | ENSOARG00020015231 | PGD | protein coding | phosphogluconate dehydrogenase [Source: NCBI gene; Gene ID:443541] |
|  |  |  | NA | APITD1 (CENPS/ LOC101114968) | protein coding | centromere protein S [Source: NCBI gene; Gene ID:101114968] |
|  |  |  | ENSOARG00020015065 | DFFA | protein coding | DNA fragmentation factor subunit alpha [Source: NCBI gene; Gene ID:101114715] |
|  |  |  | ENSOARG00020015439 | KIF1B | protein coding | kinesin family member 1B [Source: NCBI gene; Gene ID:101115220] |
|  |  |  | NA | LOC105606447 | pseudogene | 60S ribosomal protein L39-like [Source: NCBI gene; Gene ID:105606447] |
|  |  |  | ENSOARG00020016855 | UBE4B | protein coding | ubiquitination factor E4B [Source: NCBI gene; Gene ID:101113088] |
|  |  |  | NA | RBP7 | protein coding | retinol binding protein 7 [Source: NCBI gene; Gene ID:101115811] |
|  |  |  | ENSOARG00020017395 | LZIC | protein coding | leucine zipper and CTNNBIP1 domain containing [Source: NCBI gene; Gene ID:101116332] |
|  |  |  | ENSOARG00020017352 | NMNAT1 | protein coding | nicotinamide nucleotide adenylyltransferase 1 [Source: NCBI gene; Gene ID:101116072] |
|  |  |  | ENSOARG00020017441 | CTNNBIP1 | protein coding | catenin beta interacting protein 1 [Source: NCBI gene; Gene ID:101116924] |
|  |  |  | ENSOARG00020017935 | PIK3CD | protein coding | phosphatidylinositol-4,5-bisphosphate 3-kinase catalytic subunit delta [Source: NCBI gene; Gene ID:101117348] |
|  |  |  | ENSOARG00020017483 | CLSTN1 | protein coding | calsyntenin 1 [Source: NCBI gene; Gene ID:101113351] |
|  |  |  | ENSOARG00020018111 | TMEM201 | protein coding | transmembrane protein 201 [Source: NCBI gene; Gene ID:101113612] |
|  |  |  | ENSOARG00020018253 | SLC25A33 | protein coding | solute carrier family 25 member 33 [Source: NCBI gene; Gene ID:100147780] |
|  |  |  | NA | LOC105606444 | ncRNA | uncharacterized LOC105606444 [Source: NCBI gene; Gene ID:105606444] |
|  |  |  | NA | LOC101113870 | pseudogene | developmental pluripotency-associated protein 2-like [Source: NCBI gene; Gene ID:101113870] |
|  |  |  | NA | LOC105606443 | ncRNA | uncharacterized LOC105606443 [Source: NCBI gene; Gene ID:105606443] |
|  |  |  | ENSOARG00020018334 | SPSB1 | protein coding | splA/ryanodine receptor domain and SOCS box containing 1 [Source: NCBI gene; Gene ID:101117602] |
|  |  |  | NA | LOC105616528 | ncRNA | uncharacterized LOC105616528 [Source: NCBI gene; Gene ID:105616528] |
|  |  |  | ENSOARG00020018357 | H6PD | protein coding | hexose-6-phosphate dehydrogenase/glucose 1-dehydrogenase [Source: NCBI gene; Gene ID:101114124] |
|  |  |  | ENSOARG00020018439 | GPR157 | protein coding | G protein-coupled receptor 157 [Source: NCBI gene; Gene ID:105606440] |
|  |  |  | ENSOARG00020018688 | SLC2A5 | protein coding | solute carrier family 2 (facilitated glucose/fructose transporter), member 5 [Source: NCBI gene; Gene ID:443507] |
|  |  |  | ENSOARG00020018978 | CA6 | protein coding | carbonic anhydrase 6 [Source: NCBI gene; Gene ID:101117861] |
